# A Garden Colony for Consumptives

**Published:** 1916-04-29

**Authors:** 


### A Garden Colony for Consumptives.

### INTERESTING EXPERIMENT AT LEICESTER.

Leicester Sanitary Committee is about to enter upon an experiment in the treatment of consumptives which will be followed with great interest throughout the country. Some time ago the Borough Insurance Committee asked them to consider the advisability of establishing a garden colony for such patients, owing to the Darley Dale arrangement having fallen through. The Derbyshire scheme enabled the Insurance Committee to send a number of consumptives to engage in light garden work, and so successful was the experiment from a health point of view, that the Committee, on being informed that the proprietor of the gardens in Darley Dale could no longer continue the arrangement, sought the help of the Sanitary Committee in establishing a colony near Leicester.

The Sanitary Committee, in turn, communicated with the Estates Committee, with the view of utilising for the purpose the extensive gardens of Leicester Frith, which are about to come under their control. There are many acres of garden ground, orchards, and glass at the Frith, and the Sanitary Committee offered to bring these under cultivation by the consumptive labour they were able to supply. The Estates Committee readily fell in with the suggestion, and the scheme will be entered upon almost immediately. An expert gardener will be engaged to give instruction, and it is hoped that every patient who is in a physical condition to do the work, after having received institutional treatment, will remain at least one month at the garden colony.

A special committee has been appointed, with Councillor Wilford at its head, to administer the scheme. It is hoped so to train some of the men that it will not be necessary for them to return to their indoor trades as a means of livelihood, but that they will become sufficiently proficient in gardening to earn their living by it. A start is to be made at once. Dr. Kellick Millard, the medical officer of health, is enthusiastic over the scheme, believing that through it excellent results will be obtained.

For Matrons' and Sisters' Appointments see page vi.

